# Psychiatric disorders and urbanization in Germany

**DOI:** 10.1186/1471-2458-8-17

**Published:** 2008-01-17

**Authors:** Jack Dekker, Jaap Peen, Jurrijn Koelen, Filip Smit, Robert Schoevers

**Affiliations:** 1 JellinekMentrum Mental Health Institute. Klaprozenweg 111. P.O. Box 75848. 1070 AV Amsterdam, The Netherlands; 2 Trimbos Institute, Da Costakade 45. P.O. Box 725. 3500 AS Utrecht, The Netherlands; 3 Vrije Universiteit, Department of Clinical Psychology. Van der Boechorstraat 1. 1081 BT Amsterdam, The Netherlands; 4 Vrije Universiteit, EMGO. Van der Boechorstraat 1. 1081 BT Amsterdam, The Netherlands

## Abstract

**Background:**

Epidemiological studies over the last decade have supplied growing evidence of an association between urbanization and the prevalence of psychiatric disorders. Our aim was to examine the link between levels of urbanization and 12-month prevalence rates of psychiatric disorders in a nationwide German population study, controlling for other known risk factors such as gender, social class, marital status and the interaction variables of these factors with urbanization.

**Methods:**

The Munich Composite International Diagnostic Interview (M-CIDI) was used to assess the prevalence of mental disorders (DSM-IV) in a representative sample of the German population (N = 4181, age: 18–65). The sample contains five levels of urbanization based on residence location.

The epidemiological study was commissioned by the German Ministry of Research, Education and Science (BMBF) and approved by the relevant Institutional Review Board and ethics committee. Written informed consent was obtained for both surveys (core survey and Mental Health Supplement). Subjects did not get any financial compensation for their study participation.

**Results:**

Higher levels of urbanization were linked to higher 12-month prevalence rates for almost all major psychiatric disorders (with the exception of substance abuse and psychotic disorders). The weighted prevalence percentages were highest in the most urbanized category. Alongside urbanization, female gender, lower social class and being unmarried were generally found to be associated with higher levels of psychopathology. The impact of urbanization on mental health was about equal (for almost all major psychiatric disorders) in young people and elderly people, men and women, and in married and single people. Only people from a low social class in the most urbanized settings had more somatoform disorders, and unmarried people in the most urbanized settings had more anxiety disorders.

**Conclusion:**

Psychiatric disorders are more prevalent among the inhabitants of more urbanized areas. probably because of environmental stressors.

## Background

In general, admission rates for mental disorders are higher in urban areas than in rural areas [1-7]. In a nationwide study, Dekker *et al*. [6] found that, across the Netherlands, admission rates were twice as high in the most highly urbanized municipalities than in the least urbanized municipalities. Similar urban/rural differences have also been found for the incidence of psychosis in Sweden and Denmark [8,9].

These urban/rural differences are also reflected in psychiatric morbidity rates. In an epidemiological survey in the Netherlands. Bijl *et al*. [10] found an urban/rural difference in the total annual prevalence figures for psychiatric disorders in the population as a whole. This difference was also found for mood disorders. substance-induced disorders and psychotic symptoms, but not for anxiety disorders [11,12].

It is difficult to give an unequivocal explanation for the robust urban-rural differences found in the Netherlands. The two main hypotheses used in the field are the "breeder hypothesis" and the "drift hypothesis" [13]. The first hypothesis is that people in highly urbanized communities suffer from psychiatric syndromes because of environmental stressors, such as a lack of social cohesion, restricted living space, over-stimulation, low-quality housing and higher levels of crime [14-16]. The second explanatory hypothesis – the "drift hypothesis" – assumes that selective migration may take place, resulting in a concentration of the mentally ill in more urbanized environments. Although concentrations of, in particular, schizophrenic patients in deprived inner-city areas have often been documented [17,18]. evidence about the drift process within cities is sparse [19]. The number of available studies, and therefore evidence, about urban-rural drift is also sparse [20,21].

In addition to urbanization, other risk factors are gender, socio-economic class and marital status [10,22]. Depressive, anxiety and somatoform disorders are more often found in women, and substance abuse disorders are more prevalent in men [22-24]. Higher socio-economic class and being married/living with a partner are protective factors for psychiatric disorders [25]. In the adult population. the influence of age as a risk factor is less clear [26]. It is not currently known whether urbanization, gender, socio-economic classes, marriage and age may be interrelated. The interaction of urbanization with these factors has rarely been investigated.

This study examines the influence of all these associated factors on psychiatric morbidity. and their possible interrelation. This area was also studied in the most recent comprehensive nationwide morbidity and health survey in a major European country conducted in 1999 in Germany by Jacobi *et al*. [27]. Our first hypothesis is that morbidity increases with increasing levels of urbanization in Germany (as in the Netherlands). Our second hypothesis is that, after correction for distinct factors such as gender, social class, age and marriage, urbanization is still significantly related with psychiatric disorders, and that this link may also be present in interaction with gender, social class, age and marriage.

## Method

### Design

The aims. design and methods were recently described in greater detail in a separate publication [27]. In short, the aim of the 1998 German Health Interview and Examination Survey (GHS) was to describe the prevalence of somatic and mental disorders in the adult German population [22]. Mental disorders were assessed in the GHS in a sub-sample of its core survey (GHS-CS). The core survey covered a range of medical and social assessments with a response rate of 61.4% (n = 7124). The data for mental disorders were gathered using a two-stage design. The first stage entailed the administration of a screening questionnaire for mental disorders (the 12-item screening questionnaire CID-S) at the end of the medical examination. The second stage involved structured interviews with all the participants who had screened positive for a mental disorder and a random sample of 50% who had screened negative with the CID-S. The conditional response rate was 87.6% (n = 4181). As a result of the two-stage sampling design, data were first weighted to reflect the screened-positive/screened-negative sampling scheme (with Stata, version 7.0). The weighting scheme also accounts for non-response according to age, gender and geographic location [18]. The weighted results can be regarded as representative for the German non-institutionalized adult population from 18 to 65 years [22]. Detailed information about design and sampling is provided elsewhere [22,27-30].

Psychopathological and diagnostic assessments were based on the computer-assisted version of the Munich Composite International Diagnostic Interview (DIA-X/M-CIDI) [30,31], a modified version of the World Health Organization CIDI [32] covering a wider range of DSM-IV mental disorders than previous studies.

Unlike previous versions of the CIDI, the study version focuses strictly on the assessment of 12-month symptoms and disorders. The standard CIDI lifetime assessment was only performed when lifetime information was necessary for the evaluation of current diagnoses (e.g. mood disorders).

The psychometric properties of the CIDI were found to be acceptable to very good [33-35]. The interviewers (N = 24, mostly psychologists who had already worked in other CIDI studies) had received a three-day CIDI training course for the GHS-MHS, and CIDI refresher courses every three months throughout the field period. They conducted 174 interviews on average in eight sampling units and were closely monitored and provided with feedback by trained M-CIDI clinical editors who regularly checked all interviewers using a standard procedure. In a final quality control, only eight interviews had to be eliminated due to missing or inconsistent data sets [27].

The following DSM-IV mental disorders are covered here: schizophrenia and possible psychotic disorders (screening without further differential diagnosis); substance use disorders (dependence and abuse of alcohol, illicit substances); mood disorders (unipolar and bipolar); anxiety disorders (including obsessive compulsive disorder; without PTSD); somatoform disorders (including the abridged somatization syndrome; without conversion and body dysmorphic disorder) [36].

### Assessment of urbanization

In this study. five levels of urbanization were defined on the basis of two variables. The first variable was the number of inhabitants (<2000, 2000–4999, 5000–19999, 20000–49999, 50000–99999, 100000–499999, >500000 inhabitants). The second variable was a breakdown of the more urbanized municipalities into 'centres' and suburban' areas. A combination of both variables was used to define the level of urbanization of the place of residence: 1. very rural municipalities (<5000 inhabitants); 2. rural (5000–20.000 inhabitants); 3. urban-I (municipalities with 20.000–100.000 inhabitants and the suburbs of the municipalities with 100.000–500.000); 4. urban-II (centres of the municipalities with 100.000–500.000 inhabitants and the suburbs of the municipalities >500.000); and 5. urban-III (centres of the municipalities >500.000 inhabitants). These categories were described as: very rural, rural, urban, very urban, extremely urban. They included 675, 673, 958, 779 and 1096 of the respondents of the study respectively.

The adopted social class index (Winkler-Schicht Index) [37] was used to classify respondents according to the information they provided about their level of education, current job, and net household income [22]. This variable was dichotomized into lower class (20%), and middle and upper class. Marital status was divided into married (64%), and not or no longer married. Age was dichotomized into younger than 40 years of age, and above 40 years of age (52%).

The demographic characteristics of the weighted sample by degree of urbanization are presented in Table 1. The weighted demographic sample characteristics of the categories of urbanization were compared using chi-square testing.

**Table 1:** Demographic characteristics of the weighted sample (n = 4181)

		Extremely urban		Very urban		Urban		Rural		Very rural		Chi-square test	
		N	%	N	%	N	%	N	%	N	%	F	p
Gender	Female	616	52.7	418	49.2	450	50.6	307	46.5	288	46.9	9.32	.053
	Male	553	47.3	431	50.8	438	49.4	354	53.5	326	53.1		
Age	>=40 years	622	53.3	432	50.9	457	51.5	308	46.6	303	49.3	8.22	.084
	<40 years	546	46.7	417	49.1	431	48.5	353	53.4	312	50.7		
Marital status	Married	676	59.3	522	62.6	588	68.1	427	65.2	412	68.0	22.89	.000
	Not married	464	40.7	312	37.4	275	31.9	228	34.8	193	32.0		
Socioeconomic class	Low	210	18.5	170	20.4	153	17.6	132	20.2	118	19.5	2.93	.570
	Middle/high	925	81.5	663	79.6	714	82.4	522	79.8	487	80.5		

The most urbanized category has the largest proportion of women (52.7%), people aged 40 years and older (53.3%) and unmarried people (40.7%). However, only the distribution of marital status differs significantly according to degree of urbanization. The proportion of people in the low socioeconomic class does not vary greatly according to degree of urbanization.

### Statistical analyses

Firstly. the weighted percentages were determined for the different levels of urbanization, together with the standard errors. The data were weighted for age, gender and region in accordance with national administration statistics [27]. Using logistic regression (SPSS), the significance of the urbanization factor was determined for any psychiatric disorder, and for each of the disorders separately. Comorbidity rates (one or more disorders) were also calculated for the five main diagnostic categories (mood, anxiety, somatoform, substance abuse and psychotic disorders). Differences were subjected to chi-square testing.

Secondly, logistic regression (method backward with Stata) was used to calculate the odds ratios (ORs) for the different urbanization categories (the reference category is that of the most rural municipalities). In the same analysis odds ratios were calculated for gender (the reference group is men), social class (the reference group is the middle/upper class), for marriage (the reference group is not married) and for age (the reference group is younger than 40 years of age).

Thirdly, we looked at interactions between urbanization and gender, socioeconomic class, marriage and age. We dichotomized all variables, mainly to simplify the interpretation of the interaction terms. Because of the significant differences between the two most urbanized municipalities and the other categories (see Table 2) we made a group with very urban and extremely urban municipalities and a group with all other municipalities. In addition, we established interaction variables. The first of these was between gender and urbanization: women in the most urbanized municipalities and all other persons. The second was between socioeconomic class and urbanization: persons from the lowest class in the most urbanized municipalities and all other persons. The third was between marriage and urbanization: the unmarried people in the most urbanized municipalities and all other persons. The fourth was between age and urbanization: the elderly (>40 years (accounting for about 50% of the sample)) in the most urbanized municipalities and all other persons. (In all three cases, the group named second was the reference group). Logistic regression (method backward with Stata) was used to calculate the odds ratios (OR's). The OR's are presented for the significant risk factors for all the main diagnosis categories.

**Table 2:** Prevalence of psychiatric disorders in the last 12 months in adults (18–65 years of age) in Germany in relation to urbanization in 1999 (weighted percentages with standard error)

	Urbanization										
	Extremely urban		Very Urban		Urban		Rural		Very Rural		
	N = 1169		N = 849		N = 888		N = 661		N = 614		
	%	Se	%	Se	%	Se	%	Se	%	Se	p for trend
any mood disorders	15.2% (178)	1.1%	12.4% (105)	1.2%	10.6% (94)	1.0%	9.8% (65)	1.1%	9.3% (57)	1.1%	.000
major depressive disorder	11.1% (130)	0.9%	7.7% (65)	1.0%	6.9% (61)	0.8%	7.5% (50)	1.0%	6.8% (42)	1.0%	.001
any bipolar disorder	1.2% (14)	0.3%	1.2% (10)	0.4%	0.5% (4)	0.2%	0.5% (3)	0.3%	0.3% (2)	0.2%	- *
dysthymia	4.8% (56)	0.6%	5.4% (46)	0.8%	4.6% (41)	0.7%	3.7% (24)	0.7%	3.2% (20)	0.7%	.047
any anxiety disorders	16.9% (198)	1.1%	12.6% (107)	1.2%	14.8% (131)	1.1%	14.3% (95)	1.4%	12% (74)	1.3%	.024
social phobia	2.6% (30)	0.5%	2% (17)	0.5%	1.5% (13)	0.4%	2.5% (17)	0.6%	1.1% (7)	0.4%	.086
any simple phobia	7.9% (92)	0.8%	7.2% (61)	0.9%	7.3% (65)	0.8%	7.6% (50)	1.0%	8.2% (50)	1.1%	.895
generalized anxiety disorder	2.1% (25)	0.4%	1.3% (11)	0.4%	1.5% (13)	0.4%	1.3% (9)	0.4%	0.9% (6)	0.4%	.065
obsessive compulsive disorder	0.9% (11)	0.3%	1.1% (9)	0.4%	0.8% (7)	0.3%	0.4% (3)	0.3%	0.1% (1)	0.1%	- *
panic disorder with/without agoraphobia	2.7% (32)	0.5%	2.6% (22)	0.6%	2.4% (21)	0.5%	2.4% (16)	0.6%	1.3% (8)	0.4%	.098
any somatoform disorder/syndrome	13.7% (160)	1.0%	13.5% (115)	1.2%	9.7% (86)	1.0%	8.2% (54)	1.1%	7.4% (45)	1.0%	.000
SSI4.6	5.6% (65)	0.7%	5.9% (50)	0.8%	3.3% (29)	0.6%	2.9% (19)	0.6%	2.8% (17)	0.6%	.000
pain disorder	9.8% (115)	0.9%	9.7% (82)	1.1%	7.6% (67)	0.9%	6.5% (43)	1.0%	5.4% (33)	0.9%	.000
any substance disorder	5.1% (60)	0.7%	3.7% (31)	0.7%	3.6% (32)	0.6%	4.9% (32)	0.8%	5.1% (31)	0.8%	.920
alcohol abuse or dependence	4.3% (50)	0.6%	3.8% (32)	0.7%	3.1% (28)	0.6%	4.9% (32)	0.8%	4.7% (29)	0.8%	.556
alcohol dependence	3.4% (40)	0.5%	3.1% (26)	0.6%	2.3% (20)	0.5%	4.3% (28)	0.8%	4% (25)	0.8%	.386
illicit drug abuse/dependence	1% (12)	0.3%	0.5% (4)	0.3%	0.8% (7)	0.3%	0.2% (1)	0.2%	0.8% (5)	0.3%	- *
possible psychotic disorder	2.4% (28)	0.5%	3.5% (30)	0.7%	2.6% (23)	0.5%	2.7% (18)	0.6%	1.4% (9)	0.5%	.208
any mental disorder	36.4% (426)	1.5%	31% (263)	1.7%	29.4% (261)	1.5%	28.3% (187)	1.7%	26.6% (163)	1.7%	.000

* Differences were not tested due to insufficient power.

The OR's for the second and third questions were calculated using the unweighted data.

These analyses were repeated for each separate diagnostic category and we were therefore unable to determine the effects of possible co-occurring disorders. As a consequence, we conducted a final analysis using ordinal logistic regression analyses, with the urbanization categories as the 'dependent' variable and the simultaneous inclusion of all diagnostic categories as 'predictor variables'. The prevalence numbers for the five main diagnostic groups were added to the model together, followed by gender, age, marital status and SES. Only significant predictors were kept in the model (p < .05). Finally, the interactions between the remaining significant predictor variables were tested to construct a final model.

## Results

Table 2 shows the weighted 12-month prevalence rates for the disorders in the different categories of urbanization. The comorbidity rates (measured over the five main diagnostic categories) are shown at the end of this table.

The 12-month prevalence of almost all disorders (except for substance abuse disorders and psychotic disorders and simple phobia) increases significantly and linearly with the level of urbanization (determined by the trend analysis of logistic regression statistics). This linearity was a trend only for social phobia. generalized anxiety disorders and panic disorder (0.05 < p < 0.10).

The proportion of people with two or more disorders increases with the level of urbanization from 25.6% to 34.0%. However. this gradient is not significant.

Table 3 presents only the OR's for the significant risk factors for all the main diagnosis categories.

**Table 3:** Urbanization, gender, social class, marriage and age as predicting variables for psychiatric disorders in last 12 months in adults (18–64 years) in Germany in 1999

**Any disorder**	Odds Ratio	Std. Err.	z	P > z	[95% Conf. Interval]	
rural	1.04	0.13	0.36	0.72	0.83	1.32
urban	1.20	0.13	1.64	0.10	0.97	1.49
very urban	1.20	0.14	1.60	0.11	0.96	1.51
extreme urban	1.43	0.15	3.32	0.00	1.16	1.76
Sex	1.71	0.12	7.93	0.00	1.50	1.96
Social class	1.34	0.11	3.53	0.00	1.14	1.58
Marriage	1.45	0.10	5.32	0.00	1.26	1.66
**Mood**						
rural	1.15	0.21	0.80	0.43	0.81	1.64
urban	1.33	0.22	1.72	0.09	0.96	1.83
very urba	1.53	0.25	2.58	0.01	1.11	2.12
extreme urban	1.66	0.26	3.25	0.00	1.22	2.25
Sex	1.90	0.19	6.55	0.00	1.57	2.30
Social class	1.40	0.15	3.08	0.00	1.13	1.73
Marriage	1.65	0.17	4.93	0.00	1.35	2.01
Age	1.22	0.12	2.03	0.04	1.01	1.49
**Anxiety**						
rural	1.10	0.16	0.63	0.53	0.82	1.47
urban	1.17	0.16	1.14	0.25	0.89	1.53
very urba	0.90	0.13	-0.69	0.49	0.67	1.21
extreme urban	1.28	0.17	1.85	0.07	0.99	1.66
Sex	2.27	0.20	9.20	0.00	1.91	2.71
Social class	1.47	0.14	3.94	0.00	1.21	1.78
**Somatoform**						
rural	0.97	0.19	-0.17	0.86	0.66	1.41
urban	1.27	0.22	1.40	0.16	0.91	1.77
very urban	1.72	0.29	3.21	0.00	1.24	2.40
extreme urban	1.69	0.27	3.24	0.00	1.23	2.31
Sex	1.95	0.20	6.49	0.00	1.59	2.38
Social class	1.25	0.14	1.95	0.05	1.00	1.57
**Abuse **						
Sex	0.23	0.04	-8.35	0.00	0.16	0.32
Social class	1.61	0.27	2.83	0.01	1.16	2.25
Marriage	2.42	0.41	5.15	0.00	1.73	3.38
Age	0.60	0.10	-2.98	0.00	0.42	0.84
**Psychotic**						
Social class	1.43	0.31	1.65	0.10	0.94	2.18
Marriage	1.40	0.27	1.73	0.08	0.96	2.04

As could be expected on the basis of the literature, gender, social class and social status were significantly associated with almost all the main categories in the expected ways. Women, people from lower social class and unmarried people had more disorders (although women had fewer abuse disorders). Elderly people had more mood disorders and fewer abuse disorders. After correction for these widespread risk factors, urbanization (especially the extremely urban category) continued to be a risk factor, except for substance abuse and psychotic disorders.

Table 4 presents only the OR's for the significant risk factors for all the main diagnosis categories. In this analysis, the risk factors and the interaction variables were all dichotomized (see method).

**Table 4:** Urbanization, gender, social class, marriage, age and their interactions as predicting variables for psychiatric disorders in last 12 months in adults (18–64 years) in Germany in 1999

**Any disorder**	Odds Ratio	Std. Err.	z	P > z	[95% Conf. Interval]	
Urbanization	1.22	0.08	2.91	0.00	1.07	1.39
Gender	1.72	0.12	7.96	0.00	1.50	1.96
Social class	1.33	0.11	3.43	0.00	1.13	1.56
Marriage	1.46	0.10	5.37	0.00	1.27	1.67
**Mood**						
Urbanization	1.36	0.13	3.33	0.00	1.14	1.64
Gender	1.90	0.19	6.56	0.00	1.57	2.30
Social class	1.39	0.15	3.01	0.00	1.12	1.72
Marriage	1.66	0.17	4.99	0.00	1.36	2.02
Age	1.23	0.12	2.07	0.04	1.01	1.49
**Anxiety**						
Interaction marriage and urbanization	1.28	0.13	2.41	0.02	1.05	1.57
Gender	2.28	0.20	9.23	0.00	1.91	2.72
Social class	1.43	0.14	3.66	0.00	1.18	1.74
Somatoform						
Urbanization	1.41	0.15	3.27	0.00	1.15	1.73
Gender	1.94	0.20	6.45	0.00	1.59	2.38
Interaction somatoform and urbanization	1.56	0.24	2.89	0.00	1.15	2.11
**Abuse**						
Age	0.60	0.10	-2.98	0.00	0.42	0.84
Gender	0.23	0.04	-8.35	0.00	0.16	0.32
Social class	1.61	0.27	2.83	0.01	1.16	2.25
Marriage	2.42	0.41	5.15	0.00	1.73	3.38
**Psychotic**						
Marriage	1.48	0.28	2.08	0.04	1.02	2.14

In this analysis also, urbanization remains a risk factor alongside the other risk factors for almost all psychiatric disorders. Other than this general effect, urbanization interacted only with anxiety and somatoform disorders. Unmarried people in the most urbanized cities had more anxiety disorders compared with all other people. People from the lower socio-economic class in the most urbanized cities had more somatoform disorders than all other people. No interaction effects were found between urbanization and gender or between urbanization and age.

Table 5 presents only the OR's for the significant risk factors of the stepwise (p < .05) ordinal regression for all the main diagnosis categories, the other risk factors and the interaction terms.

**Table 5:** Ordinal regression model predicting level of urbanization (5 levels)

	OR (95% CI)	p
Affective disorder	1.28 (1.09–1.51)	.003
Somatoform disorder	1.23 (1.01–1.49)	.039
Marital status – Not married	1.35 (1.20–1.52)	.000
SES – Low SES	0.78 (0.67–0.90)	.001
Som. disorder * SES – Som. Disorder and low SES	1.78 (1.19–2.67)	.005

Affective disorders and somatoform disorders are more often found in areas with high levels of urbanization. Independently of this, unmarried people are more often found in urban areas, while numbers of people with low SES are lower in urban areas. Furthermore, an interaction was found between somatoform disorders and SES, implying that people with low SES who are suffering from a somatoform disorder are more often found in urban areas. Table 4 showed that this interaction was significant, as was the interaction between marriage and urbanization. Adding the latter interaction term did not improve the model.

## Discussion

This current study set out to establish an association between level of urbanization and psychiatric morbidity in a nationwide German epidemiological survey. It was shown that – as in the Netherlands – higher levels of urbanization are related to higher 12-month prevalence rates of almost all psychiatric disorders. The weighted prevalence percentages of the major disorders were highest in the most highly urbanized category. For at least one disorder, the most pronounced prevalence ratio – between the very rural category and the extreme urban category – was 1:1.5 in this study. This is similar to the ratio found in the Dutch study [11].

There was no relation between urbanization on the one hand and substance abuse disorders on the other. This is in accordance with other European urban-rural comparison studies, such as the recent ESEMeD study by Kovess-Masféty et al. [38].

The fact that we found no relation between urbanization and psychotic disorders concurs with the Nemesis study in the Netherlands [11], in which – as in this study – the one-year prevalence rate was chosen as the point of reference. One of the most likely explanations for the fact that no association was detected is that the absolute number of psychotic patients is low in a population survey among 4.700 inhabitants. If lifetime prevalence or psychotic symptoms had been chosen as a reference point instead. an association between psychosis and urbanization may have been found. For example, Van Os *et al*. [12] found a relation between psychotic symptoms and the lifetime prevalence of psychotic disorders, and between psychotic symptoms and urbanization. Due to these differences in the conceptualization of psychosis, it cannot be concluded that our findings are at odds with the Van Os study.

An important difference with the Dutch study [11] is the possibly less accurate assessment of the degree of urbanization in the present study. In the Dutch study, urbanization for a municipality was determined by taking the average for the address density of all the individual addresses in a municipality. In this study, a more simple measure was used combining the number of inhabitants and dividing municipalities into centre and suburbs. This measure is possibly less accurate than the address density method employed in the study by Peen *et al*. [11]. A further limitation of the present study is that homeless and institutionalized people are not represented in the sample. An underestimation of the prevalence of psychopathology in the more urban municipalities is therefore a distinct possibility. A final shortcoming is that in the present study. possible differences in response rates as a function of urbanization were not addressed. Even though the conditional response rate in the second stage was rather high (86%). the core survey response rate of the first stage is lower (64%). In the first stage examinations were conducted at sampling units in the second stage mental health interviews were conducted in the respondents' homes. The possibility cannot therefore be excluded that a selection occurred in the first stage as a consequence of a higher burden of participation due to larger travelling distances in rural areas.

In addition to urbanization, female gender, lower social class and being unmarried are generally found to be associated with higher levels of psychopathology. In this study, most disorders were more prevalent among women. The exception was substance abuse, which is seen more often in men. Lower social class is a risk factor for almost all disorders. Interestingly, the prevalence of psychiatric disorders is higher in unmarried people. A possible explanation is that marriage is often concurrent with good interpersonal relationships and consequently with better psychological health, while divorced or unmarried people have more difficulty in building enduring and stable intimate relationships [39]. Age in the adult population is not a factor of pronounced importance. These results are in accordance with other epidemiological studies in the Netherlands [10] and in other European countries [38].

Turning to the interaction of isolated risk factors with degree of urbanization, only two findings were significant. People from a low social class in the most urbanized settings had more somatoform disorders. and unmarried people in the most urbanized settings had more anxiety disorders. It would appear that. for young or elderly people, men or women, and married or unmarried people, urbanization has an equal impact on mental health. Urbanization was linked to a higher prevalence of somatoform disorders among people with lower SES. This finding concurs with the breeder hypothesis, which states that the inhabitants of more urban settings have more psychiatric disorders because of environmental stressors. Typical stress factors (i.e. those frequently mentioned in the literature) are the scarcity of social cohesion and/or control [40], limited living space, over-stimulation, a lot of low-quality homes, and a higher rate of criminality [41]. Assuming a high degree of lifetime stability of urban exposure [12] these results concur with the conclusion arrived at by Van Os *et al*. [12], that 'high levels of deprivation and social isolation and low levels of social capital in the urban environment may enhance the development of "at risk" mental states'.

## Conclusion

This study confirms that psychiatric disorders are more common in more urbanized areas in Germany. Alongside urbanization, female gender, lower social class and being unmarried were generally found to be associated with higher levels of psychopathology. The impact of urbanization on mental health was about equal (for almost all major psychiatric disorders) in young people and elderly people, men and women. and in married and single people. Only people from a low social class in the most urbanized settings had more somatoform disorders, and unmarried people in the most urbanized settings had more anxiety disorders. The urban-rural differences found may be related to environmental risk factors, although drift processes cannot be ruled out.

## Competing interests

The author(s) declare that they have no competing interests.

## Authors' contributions

JD, JP, JK and RS wrote the Introduction, Method and Discussion section.

JP did after consultation of FS with JD the statistical analyses and wrote with JD and FS the Result section.

All authors have given final approval of the version to be published.

JP arranged with F. Jacobi (TU Dresden) the data of the German epidemiological study for these analyses.

## Pre-publication history

The pre-publication history for this paper can be accessed here:


